# The Role of Inflammation in Crohn's Disease Recurrence after Surgical Treatment

**DOI:** 10.1155/2020/8846982

**Published:** 2020-12-26

**Authors:** B. Sensi, L. Siragusa, C. Efrati, L. Petagna, M. Franceschilli, V. Bellato, A. Antonelli, C. Arcudi, M. Campanelli, S. Ingallinella, A. M. Guida, A. Divizia

**Affiliations:** ^1^Department of Surgery, Tor Vergata University of Rome, Viale Oxford 81, 00133 Rome, Italy; ^2^Department of Gastroenterology, Ospedale Israelitico, Rome, Italy

## Abstract

**Introduction:**

Postoperative recurrence after surgery for Crohn's disease (CD) is virtually inevitable, and its mechanism is poorly known.

**Aim:**

To review the numerous factors involved in CD postoperative recurrence (POR) pathogenesis, focusing on single immune system components as well as the immune system as a whole and highlighting the clinical significance in terms of preventive strategies and future perspectives.

**Methods:**

A systematic literature search on CD POR, followed by a review of the main findings.

**Results:**

The immune system plays a pivotal role in CD POR, with many different factors involved. Memory T-lymphocytes retained in mesenteric lymph nodes seem to represent the main driving force. New pathophysiology-based preventive strategies in the medical and surgical fields may help reduce POR rates. In particular, surgical strategies have already been developed and are currently under investigation.

**Conclusions:**

POR is a complex phenomenon, whose driving mechanisms are gradually being unraveled. New preventive strategies addressing these mechanisms seem promising.

## 1. Introduction

Crohn's disease (CD) is a chronic inflammatory bowel disease (IBD) affecting 1.5-213 persons per 100,000, depending on geographical location and lifestyle. Management of CD has become increasingly sophisticated and complex, yet no definitive cure exists. In fact, although the majority of CD patients will be treated surgically at least once in their lives, postoperative recurrence (POR) of the disease has proven virtually inevitable [1–5]. Endoscopic evidence of CD recurrence in the preanastomotic ileum is found in approximately 60% [6] of patients at six months and 80-90% at 12 months [7] from the index operation [8, 9]. Endoscopic POR is not a synonym for clinical recurrence although generally the former heralds the latter [10]. In particular, 15-30% of patients will complain of florid CD symptomatology at 12 months [11–14], 20-40% at 24 months, and so on [15]. Re-do surgery occurs in up to 50% of patients with long-term follow-up [16].

The precise pathogenesis of postoperative recurrence is not known although many studies have shed light on the pathways involved. Inflammation is definitely the ultimate mechanism of tissue destruction that is reinitiated in POR. A deeper understanding of recurrence mechanisms may help clinicians and surgeons refine treatment and improve outcomes.

## 2. Aim

The authors' aim is to provide a comprehensive review of the processes involved in CD POR pathogenesis.

We will analyze how each component of the immune system has a part in CD POR pathogenesis, as well as immune function as a whole, and highlight its significance in relation to initiating factors and preventive strategies.

## 3. Methods

**Figure 1:**
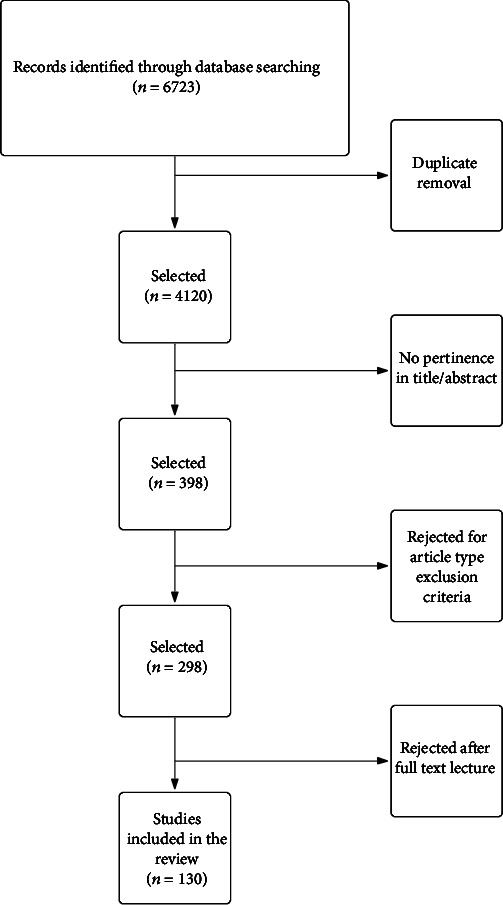
Systematic review methodology.

A systematic literature search was performed, followed by a narrative review summarizing the main findings. The systematic search was carried out on MEDLINE and Cochrane electronic databases to identify articles on CD POR. Keywords used were “Crohn recurrence,” “Crohn surgery,” and “Crohn inflammation.” The search was followed by duplicate removal and rejection of not pertinent studies or not fulfilling inclusion criteria (Figure 1). Studies included were original articles that referred to the recurrence of Crohn's disease after including randomized controlled trials, prospective and retrospective cohort studies, and case reports. Excluded article types were narrative reviews, videos, letters, conference proceedings, and abstracts. Studies that were not considered pertinent after full-text review (i.e., not addressing postsurgical recurrence pathogenesis issue specifically) were also excluded. References of all included articles were checked for additional paper identification. Papers in languages other than English were excluded from the search (Figure 1).

We use the terms “recurrence” and “POR” in the text interchangeably and “relapse” when referring to the occurrence of symptoms after a period of medically induced remission.

## 4. Pathogenesis

**Figure 2:**
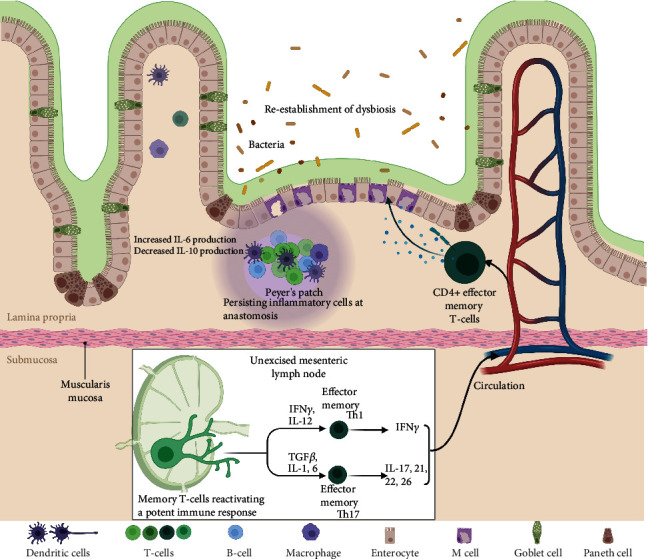
Pathogenesis of Crohn's disease recurrence. Created with https://BioRender.com

The pathogenesis of Crohn's disease is a complex one, and its precise mechanisms are still largely unknown. However, it is clear that it results from an interplay of factors such as genetics, environment, and dysbiosis, leading to immune dysregulation. Postoperative recurrence of CD is possibly an even more obscure event, yet again, it is beyond doubt that the host's immune system plays a major role. A summary of pathogenic mechanisms can be found in Table 1 and Figure 2.

## 5. Innate Immune System

The innate immune system represents the body's first line of defense. It includes physical barriers (such as cells of the epidermis or mucosa, which can also actively contribute with their secretions) and immune cells such as granulocytes, monocytes, dendritic cells, and natural killer (NK) lymphocytes. Most of these elements have been shown to be involved in CD, but little evidence exists on the innate system's role in CD POR. Nonetheless, mechanisms involved in disease pathogenesis may also be implied in disease recurrence, while others appear to be specific for POR.

### 5.1. Derangements in the Pattern Recognition Receptor and Other Genes

First, genetic analysis has identified a number of genes associated with greater odds of recurrence. The first gene to be correlated to CD has been NOD2/CARD15. This gene is an intracellular pattern recognition receptor (PRR), involved in the recognition of peptidoglycans and activation of innate immune responses. While its role in CD pathogenesis is certain, its implication in recurrence, although reasonable, is less definite and a meta-analysis [17] has failed to demonstrate a significant association [18]. A more recent study has identified instead, at multivariable analysis, an independent association between recurrence and a particular NOD2 polymorphism, rs2066844, which was not previously investigated [19]. Another gene, SMAD3, has never been linked to CD pathogenesis but was found to be an independent predictor of recurrence after ileocolic resection [20]. The authors speculate that SMD3 polymorphism may pathologically increase the scarring process after surgery and enhance fibrosis, resulting in accelerated recurrence and the need for reoperation. Finally, a recent study on 137 patients with bowel resection for CD analyzed more than 200 genetic variables [21]. Only CARD8, a gene that is highly expressed in monocytes and in the gut epithelium was independently predictive of surgical recurrence. CARD8 is a negative regulator of nuclear factor-*κβ* (NF-*κβ*), which is in turn a suppressor of apoptosis, and interacts with the inflammasome, which mediates IL-1 production and has a role in the maintenance of gut homeostasis. These functions place CARD8 as a regulator of innate and adaptive immunity. However, the exact pathways through which CARD8 enhances surgical recurrence have yet to be elucidated.

### 5.2. Matrix Metalloproteinases

Matrix metalloproteinases (MMPs) are a group of endoproteinases with proteolytic activity directed toward various matrix proteins, secreted by inflammatory cells (chiefly macrophages). Tissue inhibitors of metalloproteinases (TIMPs) regulate their activity. In the inflamed mucosa of CD, the MMP/TIMP balance is tipped in favor of MMPs. One study found a TIMP-1 variant to be correlated to surgical recurrence but not to diagnostic recurrence, implying a relatively mild- or late-onset influence [22]. Investigators have also shown that higher expression of TIMP-1 and TIMP-2 in the noninflamed mucosa after resection was protective from diagnosis of recurrence and from repeated resections. The magnitude of this effect is quite impressive: the median time to recurrence in patients with low levels of TIMP-1 was 10 years, compared to 17 years in the high expression group.

### 5.3. Cells of the Innate Immune System, Their Response to Commensal Microbiota, and Clinical Implications

Neutrophils represent the main kind of cell involved initially in acute inflammation and are the main actor in the typical cryptic abscesses of IBD. No study specifically correlates these cells to CD recurrence, but their presence in intestinal nervous plexuses has been implicated in some studies (see Immune Function as a Whole). Macrophages are very important innate immunity cells, which not only are major actors of acute inflammation but also are also involved in chronic inflammatory states and represent a major link with acquired immunity. Their role in CD pathogenesis is multifaceted and includes the production of proinflammatory cytokines (in response to inappropriate TLR stimulation), generation of granulomas, mediation of fibrosis, and disruption of intestinal epithelial barrier function [23–25]. Less is known about specific actions in POR. Their infiltration in the mucosa of the neoterminal ileum of POR-free patients suggests their driving role of inflammatory events that bring about recurrence [26]. One mechanism through which they may be involved in POR has been highlighted in experimental studies that involve CX_3_CR1^hi^ cells. These are mucosal resident mononuclear phagocytes, whose role is to capture luminal bacteria, in an effort to compartmentalize the immune response and avoid inflammation. These cells therefore represent the “second line” of defense. While previously reported to be a nonmigratory population, Diehl et al. [27] have shown in experimental models that they can actually be endowed with the ability to migrate and traffic bacteria (or their antigens) to mesenteric lymph nodes, key immune inductive sites. This may well start the inflammatory process. Not only have the authors described this phenomenon but, even more interesting, they also have unveiled a tight connection with luminal microbiota. In fact, it appears that dysbiosis is a potent activator of bacterial trafficking through this route. This mechanism has not been studied in recurrence. The effects of microbiota on recurrence have been studied. Sokol et al. have demonstrated that surgery produces profound changes in ileal microbiota, reducing dysbiosis typical of CD such as increased *Gammaproteobacteria* and declined *Lachnospiraceae* and *Ruminococcaceae* [28]. The magnitude of these changes was significantly reduced in patients who developed early postoperative endoscopic recurrence, so much so that biopsies at follow-up showed that diversification in microbiota decreased in parallel with an increase in the Rutgeerts score. The authors went so far as to develop a predictive score for recurrence that was based primarily on microbiota composition at surgery. Unfortunately, this score was not applicable to patients who had undergone antibiotic therapy in the last month before surgery (a considerable fraction of CD patients) and even more importantly did not predict the outcome in patients who were administered anti-TNF preventive therapy. Putting these facts together, one might conclude that dysbiosis reestablishment after surgery induces a new bout of bacterial trafficking, immune induction, and inflammation. This model is purely speculative, and it is likely that immune memory would have a greater role than a new activation. Nonetheless, if it does have a role, two strategies would have the potential to maintain the surgery-induced changes in microbiota and slow down recurrence: probiotic administration and fecal transplantation. A multicenter randomized controlled trial investigated the effect of probiotics on early endoscopic recurrence but failed to demonstrate a protective effect [29]. It has to be said that in this study, a specific probiotic strain, *Lactobacillus johnsonii*, was used: whether other probiotics could better serve this purpose and revive this practice is unknown. Fecal transplantation brings the “probiotic strategy” to the extreme. This intervention has been extensively shown to be effective in the treatment of ulcerative colitis, but high-quality evidence lacks in CD. No randomized trial exists to date [30], and there is considerable heterogeneity in many aspects of therapy including donor selection, fecal conditions (fresh/frozen), delivery route, and antibiotic pretreatment [31–33]. Meta-analyses of cohort studies have shown that it may have a good rate of clinical remission induction [34]. To date, no study exists on the evaluation of this therapy after surgery. Given the strong rationale for its use, this may indeed be the focus of upcoming trials. Another fundamental function of innate immunity is that of dendritic cells (DCs). They are potent antigen-presenting cells whose role in CD is well established: activating site-specific Th1 and Th17 responses [35–38]. Specific populations of dendritic cells direct inflammation and dictate CD location (by inducing homing markers) [39, 40]. These cells therefore set up a potent and durable immune response that is thought to be the principal driver of CD recurrence. In this way, they drive recurrence indirectly. Whether they also play a more direct role by reviving this mechanism after resection is not known to date. Basophils have been recently found capable of serving a similar function to DCs [41].

## 6. Acquired Immune System

The acquired immune system represents the body's more refined defense machinery. It mainly relies on the action of activated lymphocytes that are capable of initiating, amplifying, and perpetuating an adaptive, antigen-specific response. Lymphocytes include B-cells responsible for humoral immunity and T-cells, which conduct the cell-mediated arm.

### 6.1. T-Cells

Cell-mediated immunity could be the real protagonist of the recurrence process. There is evidence that the mucosa of the affected bowel harbors significantly increased clonal expansions of T-cells compared to that of healthy controls [42]. Whether this clonality principally concerns CD4+ or CD8+ cells has not been consistent, as both have been implicated in different studies [42, 43]. In any case, the presence of high clonality seems to be the harbinger of endoscopic recurrence, with greater percentages of clones associated with a greater risk of recurrence [42]. Furthermore, these highly expanded clones are different from clones found in healthy tissue, supporting a pathogenic role of T-cells [44]. The T-cell receptor (TCR) repertoire seems to be different in different individuals, with no commonly targeted antigens, highlighting the singularity of each CD patient's immune response [42]. Another observation was that the TCR repertoire in the mucosa could change with surgical resection. Postoperative persistence of a TCR repertoire similar to that before resection also correlates to the development of endoscopic recurrence [42]. In one study, the investigators were capable of identifying a particular clone, highly enriched in active CD tissues, and found its presence to be correlated to the severity of endoscopic recurrence (based on Rutgeerts score) [44]. Another study identified a subset of T-cells, CD4+NKG2D+ T-cells, whose presence at the ileal mucosal margins of resection predicted endoscopic disease recurrence [43]. In fact, this subset was found to be persistent in the mucosa of patients with recurrence. CD4+NKG2D+ T-cells display high expression of tumor necrosis factor-alpha (TNF-*α*) and other proinflammatory and cytotoxic molecules. Possibly, they would be left behind in the macroscopically healthy bowel and then induce recurrence. It is unclear however how they (being already present before operation) would induce active CD only after surgery. An alternative and more consistent explanation would be that they persist in large numbers in the mesenteric lymph nodes draining the resected bowel (which instead are not removed in current CD surgery) and are then free to home back into the neoterminal ileum. In fact, lymph nodes not only are a site of immune induction but also represent the anatomical location where the majority of memory T-cells reside [45, 46]. Therefore, mesenteric lymph nodes (MLNs) may be reasonably considered the dwelling of immune memory in CD. Bsat et al. [36] have studied MLNs of CD patients in depth and found them to be enriched in Th17 memory T-cells and in particular effector memory Th17 cells (TEM). The same group has also elucidated an IL-12-dependent pathway through which these memory cells are activated in the MLN and switched to a Th1 phenotype (i.e., cytokine/molecular profile) before being recruited back to the mucosa through homing mechanisms. This places the MLN at the center of a memory-driven immune reactivation in CD and therefore, potentially, of recurrence. Enhancement of MLN in imaging studies is, in fact, predictive of active disease [47]. The so-called trafficking blocking strategy, based on the fact that lymphocytes have to home back to the, until then, healthy gut from the nodes where they reside, has been given much prominence in recent drug development with promising ongoing trials [48].

Immunological memory was also implicated by genetic studies on BACH2 (a highly conserved transcription factor that is a fundamental element in the maturation of B-lymphocytes and differentiation of T-cells, especially in regulatory T-cells) which independently correlate it to surgical recurrence [49]. The authors speculate the underlying mechanism to be the inability to memorize an appropriate (tolerant) response to luminal antigens.

### 6.2. B-Cells

The part played by B-cells in CD inflammation is less clear [50]. So far, the most studied immunoglobulins have been peripheral Anti-Neutrophil Cytoplasmic Antibodies (pANCA) and Anti-Saccharomyces cerevisiae Antibodies (ASCA). Taken together, they are good serologic markers for CD. Other antigens eliciting a humoral response in CD are Omp-C (outer bacterial membrane protein, E. coli-derived), CBir1, A4-Fla2, and Fla-X (flagellin subunits, Clostridium-derived). In one study, anti-Fla-X positivity at baseline was associated with endoscopic recurrence at 18 months while anti-Omp-C with multiple surgeries [51]. The significance and the processes underlying these findings are yet unclear. Antibodies could be inducing recurrence also through the neutralization of protective cytokine signaling. Granulocyte-Macrophage Colony-Stimulating Factor (GM-CSF) enhances innate immune responses to luminal microbial antigens, thereby confining inflammation, avoiding antigen trafficking and activation of secondary immunity, and contributing to homeostasis. Anti-GM-CSF antibodies have been recorded in patients with CD, and their titers positively correlated with a shorter time to the second surgery [52].

It is possible that serology will be eventually used to predict the postoperative course and identify patients who would profit from a more aggressive, proactive treatment strategy.

## 7. Immune Function as a Whole

Some factors that have been associated with recurrence cannot easily be categorized as “innate” or “adaptive” immunity but have to be considered affecting the immune system as a whole.

### 7.1. Cytokines

Cytokines are the signaling molecules of the immune system. They contribute to a variety of functions and are fundamental for a proper inflammatory response to take place. Some specific cytokines are known to be involved [53–59] and represent current therapeutic targets, others have been identified, and their targeting holds promise. In one study on 36 patients undergoing bowel resection, IL-6 was found to be elevated in most patients despite clinical remission, indicating persistent subclinical (systemic) inflammation through the IL-6-C-reactive protein (CRP) cascade [60, 61]. Patients with surgical recurrence of their disease had particularly high IL-6 levels, which resulted in a significant predictor of recurrence in this series. Yet this was extrapolated from only three patients, and two of them had recurred within 5 months.

Even though very early surgical recurrence is a possibility, this more often represents a complication of the first intervention or preexisting disease that was overlooked or judged nonsignificant at the first operation. Nonetheless, this study highlights the ongoing inflammation that underlies the postoperative CD course and eventually recurrence. More evidence on the subject comes from a Japanese group [62]. They took blood samples and ileal biopsies from 36 resected patients: 16 of them had clinical recurrence within 1 year. In this group, only ileal mucosal IL-6 levels correlated significantly with clinical recurrence, but not serum possible that patients with surgical recurrence are a group with more aggressive and advanced disease and therefore present high IL-6 levels systemically rather than only at the primary inflammatory site. Reduction of anti-inflammatory cytokines is another mechanism observed to correlate with endoscopic lesions after surgery. In particular, IL-10 levels in the ileal mucosa predict the development of postoperative recurrence. Different IL-10 haplotypes exist and are associated with greater or lesser production of the cytokine, yet these were not able to predict recurrence [63]. It has been therefore speculated that other local environmental factors, so far not predictable, are responsible for IL-10 production in the mucosa. RNASET2, a gene shown to be involved in enhancement of IFN*γ* production (the main cytokine involved in Th1 responses), has been observed to be associated with more severe recurrence (Rutgeerts score > 2) and, in line with this, to shorten time to repeat surgery [64]. A putative mechanism would therefore be an enhancement of adaptive cellular immunity and a quicker and more severe postoperative reestablishment of disease. Other observations come from direct clinical practice. The most used drug for the prevention of postoperative recurrence is infliximab, a monoclonal antibody directed against TNF-*α*. Infliximab is of proven benefit in this situation and is recommended by current guidelines. In particular, there is high-quality evidence from a randomized controlled trial that it reduces the incidence of endoscopic recurrence (by a half!) at one year from surgery [65]. At the same time, it did not reduce clinical recurrence in the same period. Yet, clinical recurrence occurs for the majority of patients beyond this period, and therefore, the real clinical impact of infliximab has yet to be revealed with longer-term follow-up. Moreover, there is some evidence pointing to fewer operations for CD during the anti-TNF era [66]. This may indeed have an impact on surgical recurrence rates although this is so far unproven. Finally, the effect of anti-TNF therapy on endoscopic remission may be more pronounced in patients who were previously naïve or had undergone therapy with only one anti-TNF agent [67].

### 7.2. Inflammation in Resection Margins and Intestinal Nervous Plexuses

Another analyzed factor is histologic evidence of inflammation at resection margins. Inflammation is defined in different studies in many different ways and includes inflammation of the mucosa, of the submucosa, and even of the nerve plexuses. The first reports on microscopic evidence of inflammation at resection margins have done much to shape the current concepts of surgery for CD. It was shown that the presence of features of chronic inflammation or diagnostics of CD at resection margins did not influence recurrence rates [68, 69]. This was the basis to advocate as short a resection as possible, on the macroscopically healthy bowel [70]. This approach has been the standard practice for decades and is thought to have reduced the risk of short bowel syndrome. Lately though, this subject has been revived by new studies that have given us a slightly different perspective. Poredska et al. have reported resection margin inflammation (at both the proximal and distal margins) to be related to endoscopic POR [71]. In their study, “inflammation” included signs of both the acute and chronic forms. One recent report has focused instead on active inflammation at resection margins (defined as a Geboes score > 3) and found that its presence on the colonic margin was the only histologic predictive factor for clinical POR [72]. They failed to demonstrate any correlation for other histologic factors like myenteric plexitis and granulomas. Other investigators have studied both factors widely, so far with nondefinitive results. Granulomas' presence in the histologic specimen seems to predict surgical POR and a shorter time to the second surgery in a large meta-analysis [73]. Myenteric plexitis was reported to be a risk factor for endoscopic POR in a landmark study by Ferrante et al. in 2006 [74]. More research focused on submucosal plexitis and found a similar association (again, not consistently) [75, 76]. Finally, one study incriminated increased enteric plexus glial cells [77]. The trouble to compare these studies is that most are retrospective and that they define “inflammation,” “plexitis,” etc. in different ways, as there is no consensus or grading system specific for these features in CD [78]. In fact, results are variable as to which feature is predictive as much as where to assess it and how. In general, predicting POR through a baseline histologic assessment on the index specimen is a promising approach but one that still requires confirmation and refinement before entering common clinical practice (or trials).

### 7.3. Diet

Another very interesting factor is diet. CD patients are at high risk of malnutrition, in both the active and quiescent diseases, and dietary evaluation is fundamental in improving nutritional status, thus ameliorating disease outcomes [79]. Some investigators have specifically assessed diet influence on POR. In particular, an elemental diet (ED) has been advocated to reduce bowel inflammation. Most studies on the subject come from Japan, where it is used as standard therapy, in contrast to the USA or Europe [80]. It consists mainly of amino acids, which do not possess any intrinsic antigenicity and therefore are bereft of proinflammatory capacity. While large experience has been accumulating with its use in relapses, there are relatively few studies in the postoperative setting. The major limitation to ED is that patient compliance is generally low as it can be quite distasteful. When large amounts of calories have to be taken as ED, the use of a self-inserted nasogastric tube is commonly advised. This practice has its own obvious limitations, and adherence is limited. Yamamoto et al. conducted a prospective controlled study on long-term postoperative ED in 40 patients. Patients were not randomized but arbitrarily assigned to non-ED vs. ED based on predicted compliance with the regimen. The two groups differed significantly at 1 year in terms of endoscopic remission [81]. At 5 years, clinically significant recurrence was impressively in favor of the ED group (10 vs. 45%) [82]. Surgical recurrence also showed a trend in favor of the ED group but was not significant. Another study showed similar results on endoscopic POR [83]. Despite fascinating results, they should still be considered preliminary as the number of patients was small and the study design was not devoid of bias. Today, the method has not entered common practice and has not gathered excessive interest in western countries.

## 8. Other Risk Factors

Other risk factors for POR that are not strictly related to the immune system are as follows: penetrating disease at index surgery, perianal disease [84], prior intestinal surgery extensive small bowel resection (>50 cm), and smoking [84, 85]. No clear explanation exists for any of these: probably, they simply herald a more aggressive phenotype. Smoking is undoubtedly the most studied with a higher level of evidence [86–89]. An interesting finding by Allez et al. is that smoking is associated linearly with hyperclonality of T-cells in the affected mucosa [42]. This data therefore links smoking to the actual inflammatory processes that lead to recurrence: specific CD4+ T-cell clones.

Ileocolic disease may recur more often than disease at other sites when assessed morphologically, yet it is less often symptomatic, and rates of clinical recurrence are lower [7]. One study has tried to put these elements together in a nomogram to predict early disease recurrence [90]. Interestingly, they have also included low preoperative serum albumin and “excessive perioperative inflammation” (expressed as high CRP levels). This attractive instrument however has not been widely validated so far.

## 9. Prevention

We have so far analyzed a number of risk factors for the development of CD recurrence. The usefulness of this knowledge would be to detect patients in greater peril and to use treatment strategies accordingly. This concept is already in place for what concerns postoperative drug treatment, with anti-TNF and immunosuppressants being used in patients at high risk or with signs of endoscopic POR. Other pharmaceutical targets will surely be investigated in the coming years. Different medical pathways may also prove of benefit in the future such as fecal transplantation and hematopoietic stem cell transplantation. New surgical approaches are also promising. A summary of preventive strategies may be found in Table 2.

### 9.1. Hematopoietic Stem Cell Transplantation

In the last decade, much work has been focused on one objective: resetting the entire immune system. This may be accomplished through a treatment well known to hematologists: hematopoietic stem cell transplantation (HSCT).

HSCT for CD has been inspired by two parallel phenomena. The former was the observation of prolonged (yet not indefinite) remission in patients with HSCT performed for other (hematologic) indications [91]. The latter was the success of this strategy in patients suffering from other autoimmune diseases like systemic sclerosis and systemic lupus erythematosus. Of course, given the high toll requested by allogenic HSCT, trials have used autologous HSCT. Many investigators have reported encouraging results [92]. The main drawback of this approach is the high rate of complications and mortality that persists even with the autologous form [93]. The highest level of evidence comes so far from the ASTIC trial. This trial failed to show the superiority of HSCT in achieving the primary outcome [94]. Yet, the primary outcome was an extremely ambitious one: sustained disease remission at 12 months, defined as a composite of Crohn′s Disease Activity Index (CDAI) < 150, no active treatment for at least 3 months, and no endoscopic evidence of disease (erosion/ulceration). A different analysis of data coming from the same trial, using conventional endpoints used in trials for CD, highlighted a significant benefit in the treatment arm [95]. When relapse occurs, patients tend to respond to conventional treatment to which they had become refractory prior to transplantation [96]. A new randomized trial has successively been set up, ASTIClite, using a lower dose regimen in an effort to maximize the benefit/hazard ratio [97]. No trial is currently evaluating HSCT in the postoperative setting.

### 9.2. Surgical Approach and Procedures

Surgical intervention has also been at the center of much research in an endeavor to diminish POR, but the surgical approach did not appear to have any consequence on the short- or long-term disease course [98–104]. Nevertheless, the following are currently the more promising research fields.

#### 9.2.1. Anastomosis

The anastomosis type and fashioning technique have also been the objective of many investigations. Stapled anastomosis (despite boasting lower leak rates) is not superior to handsewn in terms of recurrence rates [105]. Exactly the same considerations are true for side-to-side vs. end-to-end anastomosis [106, 107]. In an effort to reduce recurrence, some investigators have proposed new surgical techniques [108, 109]. Kono et al. published in 2011 their first report on a new anastomotic technique [108]. Their results were impressive with an enormous reduction in surgical recurrence rates at 5 years when compared with historical cohorts. The authors ascribe this magnificent effect on their particular technique, which was designed to maintain the anastomosis diameter and dimensions. It is not clear how this would then affect recurrence rates, as CD recurrence notoriously occurs in the preanastomotic neoterminal ileum, rather than at the anastomotic site. Nonetheless, their results have been reproduced in a series from a single center in the USA [110] and another from multiple centers in Japan [111]. One study [112] showed superiority against end-to-end (EE) anastomosis, yet the latter is not recommended by issued guidelines [113] due to its poorer results compared to standard latero-lateral anastomosis. Finally, a recent randomized controlled trial documented benefits in terms of 6 and 12 months of endoscopic recurrence rates and 24 months of clinical recurrence rates [114]. This study represents an enormous effort. Yet, a bias that cannot be overestimated was that the endoscopist evaluating patients at follow-up was not blinded to the procedure performed. Given the known operator dependence of the Rutgeerts score, results from this trial cannot be overly reliable. Although all reports vouch for the safety of the Kono-S anastomosis, it is technically demanding and time-consuming. To date, the literature produced so far on this interesting technique is not entirely trustworthy and doubts about its real efficacy remain.

#### 9.2.2. Strictureplasty

An alternative to resection can be strictureplasty. Its role is well defined in some cases (where its bowel-sparing benefits are relevant) and less so in others. In any case, long-term results may be comparable to resection, although this is not clear and a high-quality head-to-head comparison is lacking. The most interesting feature of strictureplasty regards the Michelassi configuration. This side-to-side isoperistaltic strictureplasty (SSIS) has been reported to induce healing of the mucosa as seen with postoperative endoscopy. The mechanisms underlying this prodigious feat are currently unknown although it has been suggested that resolution of chronic obstruction would interrupt the perpetuation of active inflammation [115–117]. Surgical recurrences after SSIS are comparable to those reported for resection, around 20-30% in the long term (5-10 years) at the strictureplasty site [118, 119]. Nonetheless, SSIS is rarely performed for its technical complexity and the infrequency of its necessity: in the same department where it was ideated, only 3% of IBD patients undergo this procedure [118].

#### 9.2.3. Mesenteric Excision

Coffey et al. have shown a significant decrease in postoperative surgical recurrence in their retrospective series of patients, by adopting a more radical, “mesentery-based” surgical approach. Despite the magnitude of reported results being impressive, the inherent limitations in study design and specific aspects of the work lend themselves to perplexity and criticism [120]. Furthermore, the rationale for its use is absolutely scarce: very little literature supports the hypothesis of a “mesentery-driven” disease [121–128]. On the contrary, some evidence exists that important proinflammatory mechanisms are unique to the mucosa and cannot be observed in the mesentery [128]. Finally, the proposed approach seems very difficult to standardized as the correct amount of the mesentery to be removed is unknown and the suggested “mesenteric disease score” to evaluate the severity of mesenteric affection probably suffers from great interobserver variability [129].

#### 9.2.4. Pathophysiological Excision

**Figure 3:**
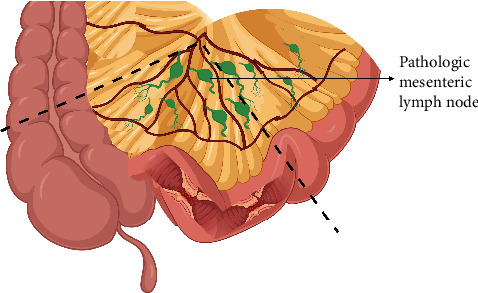
Pathophysiological excision for Crohn's (PEC). Created with https://BioRender.com

A novel approach might be that of Sica et al., presented at the Italian Group for Inflammatory Bowel Disease (IG-IBD) in 2020, but the trial is only just started (NCT04623476). This approach entails a resection of all the nodes draining the affected bowel along with the bowel itself, following a medial to lateral oncologic approach in colorectal surgery (Figure 3). The theoretical advantage is that of removing the main actors in postoperative recurrence: memory T-cells residing in the nodes. For this reason, the authors have named it “pathophysiological excision for Crohn's” (PEC). Probably, the remaining lymphocytes would have the potential to restart a full-blown syndrome. However, one interesting fact in this regard is that when lymph nodes are completely removed together with the whole bowel, the remaining circulating lymphocytes are practically incapable of reproducing CD features in a small bowel transplant [130, 131]. Nonetheless, this phenomenon (lack of proper recurrence) may be attributed to features of the transplant itself (such as different antigenicity or microbiota) or immunosuppression rather than a failure of circulating memory cells and therefore cannot be considered conclusive evidence in this sense. Nonetheless, the pathophysiological excision approach is one that is easily standardized, familiar to surgeons (from oncologic surgery), and supported by a rationale. Results of this trial could extend our knowledge and armamentarium.

## 10. Summary/Conclusion

Pathogenesis of postoperative Crohn's disease recurrence is complex and multifactorial. A primary role is surely reserved for memory T-lymphocytes. These cells migrate, following resection, from the persisting (disease-burdened) mesenteric lymph nodes to the healthy preanastomotic mucosa to perpetuate the inflammatory status. A number of diverse interventions to reduce recurrence are under evaluation, but strategies influencing the abovementioned mechanism harbor the promise of success and perhaps, one day, of cure.

## Figures and Tables

**Table 1:** Elements involved in CD recurrence.

Domain	Specific element	Putative mechanism of action	Level of evidence
Innate immune system	NOD2 gene rs2066844 polymorphism	Improper activation of the immune response (pattern recognition receptor)	Low-moderate
Innate immune system	SMAD3	Drive fibrosis after initial surgical scarring	Low
Innate immune system	CARD8	IL-1 production, immune dysregulation	Low
Innate immune system	Matrix metalloproteinases	Initiation of inflammation/fibrosis	Low-moderate
Innate immune system	Macrophages	Bacterial trafficking to mesenteric nodes	Low
Innate immune system	Dendritic cells and basophils	Set up a potent and durable acquired immune response	Low
Adaptive immune system	Effector T-cells	Bring about most recurrent inflammation	Moderate-high
Adaptive immune system	Memory T-cells	Persist after resection in mesenteric lymph nodes (and blood to a lesser degree) and conserve immunological memory	Moderate-high
Adaptive immune system	B-cells	Produce anti-GM-CSF immunoglobulins; others	Low
Immune system as a whole	Dysbiosis	Increased bacterial trafficking; others	Low
Immune system as a whole	Increased mucosal IL-6; decreased mucosal IL-10; RNASET2 polymorphism	Increase mucosal inflammation	Low
Immune system as a whole	Inflammation at resection margins	Persistence of activated cells	Low
Immune system as a whole	Myenteric and submucosal plexitis	Unknown	Low
Immune system as a whole	Diet	Stimulate the immune system through poorly understood mechanisms	Low-moderate
Other risk factors	Smoking	Unknown; may increase mucosal T-cell hyperclonality	High
Other risk factors	Penetrating disease at index surgery, perianal disease, prior intestinal surgery extensive small bowel resection (>50 cm)	Unknown	High

**Table 2:** New perspectives for recurrence prevention.

Domain	Prevention strategy	Description	Rationale
Medical	Fecal transplantation	Transfer of fecal bacteria from healthy subjects	Avoid dysbiosis reestablishment
Medical	Hematopoietic stem cell transplantation	Bone marrow ablation followed by autotransplantation of multipotent stem cells	Reset immune system
Surgical	Michelassi strictureplasty	Side-to-side isoperistaltic strictureplasty	Induce mucosal healing by resolution of chronic obstruction
Surgical	Kono anastomosis	Fashioning of antimesenteric wide lumen anastomosis	Increase the diameter and stability/durability of anastomosis
Surgical	Mesenteric excision	Excision of the associated mesentery	Removal of pathological tissue
Surgical	Pathophysiological excision	Excision of draining mesenteric lymph nodes	Removal of most memory T-cells, drivers of inflammation and recurrence

## Data Availability

The data supporting this review are from previously reported studies and datasets, which have been cited.
